# Sexual recombination is a signature of a persisting malaria epidemic in Peru

**DOI:** 10.1186/1475-2875-10-329

**Published:** 2011-10-31

**Authors:** Patrick L Sutton, Lindsay P Torres, OraLee H Branch

**Affiliations:** 1 Center for Genomics and Systems Biology, Department of Biology, New York University, New York, NY, USA; 2 Laboratorio Investigaciones Productos Naturales Antiparasitarios de la Amazonia, Universidad Nacional Amazonia Peruana, Iquitos, Peru; 3 Department of Microbiology, Division of Medical Parasitology, New York University, New York, NY, USA

## Abstract

**Background:**

The aim of this study was to consider the impact that multi-clone, complex infections have on a parasite population structure in a low transmission setting. In general, complexity of infection (minimum number of clones within an infection) and the overall population level diversity is expected to be minimal in low transmission settings. Additionally, the parasite population structure is predicted to be clonal, rather than sexual due to infrequent parasite inoculation and lack of recombination between genetically distinct clones. However, in this low transmission of the Peruvian Amazon, complex infections are becoming more frequent, in spite of decreasing infection prevalence. In this study, it was hypothesized that sexual recombination between distinct clonal lineages of *Plasmodium falciparum *parasites were altering the subpopulation structure and effectively maintaining the population-level diversity.

**Methods:**

Fourteen microsatellite markers were chosen to describe the genetic diversity in 313 naturally occurring *P. falciparum *infections from Peruvian Amazon. The population and subpopulation structure was characterized by measuring: clusteredness, expected heterozygosity (H_e_), allelic richness, private allelic richness, and linkage disequilibrium. Next, microsatellite haplotypes and alleles were correlated with *P. falciparum *merozoite surface protein 1 Block 2 (*Pf*msp1-B2) to examine the presence of recombinant microsatellite haplotypes.

**Results:**

The parasite population structure consists of six genetically diverse subpopulations of clones, called "clusters". Clusters 1, 3, 4, and 6 have unique haplotypes that exceed 70% of the total number of clones within each cluster, while Clusters 2 and 5 have a lower proportion of unique haplotypes, but still exceed 46%. By measuring the H_e_, allelic richness, and private allelic richness within each of the six subpopulations, relatively low levels of genetic diversity within each subpopulation (except Cluster 4) are observed. This indicated that the number of alleles, and not the combination of alleles, are limited. Next, the standard index of association (I_A_^S^) was measured, which revealed a significant decay in linkage disequilibrium (LD) associated with Cluster 6, which is indicative of independent assortment of alleles. This decay in LD is a signature of this subpopulation approaching linkage equilibrium by undergoing sexual recombination. To trace possible recombination events, the two most frequent microsatellite haplotypes observed over time (defined by either a K1 or Mad20) were selected as the progenitors and then potential recombinants were identified in within the natural population.

**Conclusions:**

Contrary to conventional low transmission models, this study provides evidence of a parasite population structure that is superficially defined by a clonal backbone. Sexual recombination does occur and even arguably is responsible for maintaining the substructure of this population.

## Background

The events that shape a local population structure of *Plasmodium falciparum *are not fully understood. There is a definite link between the parasite transmission rate and the amount of circulating genetic diversity within a local population; high transmission regions typically have larger pools of genetically diverse parasites, while low transmission regions typically have a limited pool of genetically diverse parasites. However, understanding how this diversity is maintained within a population is fairly unresolved due to the parasite's intricate life cycle, which has maintained both sexual and asexual phases. Unfortunately, the depth of the answer to this question is constrained by the genetic markers and molecular methods used unravel the evolutionary history by surveying the population diversity. Until deep sequencing becomes more feasible, microsatellite markers provide a relatively thorough guide to the local, and perhaps global, evolutionary history of *P. falciparum *[1], as well as other eukaryotic parasites [2-12]. Considering how genetic diversity is maintained and/or generated on a local level is a priority for advancing epidemiological applications on a molecular level by understanding how this parasite evolves in natural settings.

The contribution of sexual recombination between two unrelated *P. falciparum *clones on the population structure remains a topic for debate. Several studies have concluded that *P. falciparum *parasites are essentially clonal due to infrequent recombination between different clonal lineages, while others have concluded that sexual recombination occurs more frequently and contributes greatly to the overall population structure [13-26]. Unfortunately, measuring the contribution of population-level genetic diversity generated by sexual recombination is not straightforward, particularly in an organism like *P. falciparum*, which spends a great proportion of its life cycle in a haploid, asexual form. Several studies have investigated the contribution of genetic diversity though sexual recombination by measuring the non-random mating preference within the mosquito gut; however, data from these studies versus studies of recombination between clones from genetically distinct lineages, are incongruous [26-29].

Recombination between genetically distinct clones (or clonets) can only occur if a mosquito takes up more than one during a single blood meal or accumulates multiple clones during several interrupted feedings. The probability of a mosquito carrying multiple clones is then a function of the longevity and biting rate of the mosquito, mosquito uptake of gametocytes from different parasites clones (or clonets), and the amount of genetic diversity circulating within a parasite population [30-34]. In high transmission regions, the contribution of sexual recombination is likely to be higher and more apparent than in low transmission regions, due to increased opportunities for mosquitoes to accumulate different clones (or clonets) within the same blood meal [35-41]. Furthermore, regions of high transmission tend to have higher levels of genetic diversity with a notable decay in the linkage disequilibrium between loci, while in low transmission regions, the genetic diversity is markedly lower and disequilibrium between genetic markers is high [42]. However, even if sexual recombination between parasites of different lineages occurs at a low frequency, as is proposed in low transmission regions, the survival of the recombinants might impact the population structure and epidemiology of malaria. To test this, a longitudinal approach is necessary.

Since 2003, the Malaria Immunology and Genetics in the Amazon (MIGIA) project has been longitudinally following a low transmission (< 0.5 *P. falciparum *infections/person/year) cohort of approximately 2,000 participants located south of Iquitos, Peru in the community of Zungarococha. As this project progressed, snap-shot glances of the population-level diversity were made by using single copy, antigenic *P. falciparum *merozoite surface proteins (*Pf*msp)-1, -3, and -6. Characterization with these markers showed that the overall population-level diversity was comparable to that of other low transmission regions [41,43-45]. However, now with more than five years of longitudinal sampling data, it is clear that the population structure is far more complex than expected. A recent study, using 14 microsatellite markers scattered across 10 chromosomes, found 182 genetically distinct microsatellite haplotypes from 302 *P. falciparum *infections occurring between 2003 and 2007 [46]. Extensive population genetic analysis showed that the immigration of new clones into this low transmission population was highly improbable and could not explain the population-level diversity [46]. The investigators concluded that the level of parasite genomic diversity was effectively maintained on a temporal (2003-2007) and spatial (four networked villages) level by the loss of once frequent clones and the introduction of newly evolved ones [46].

The objective of this study was to understand how this level of diversity has been maintained temporally within this low transmission population. Across five years of longitudinal sampling, there has been a significant increase in the proportion of complex infections to single-clone infections, despite decreasing *P. falciparum *infection prevalence over time. This increased proportion of complex infections is curious, but not unique; a similar finding was observed in Thailand [39]. Such a phenomenon has the potential to set up an ecological scenario, whereby the constituents of complex infections have been provided with an increased opportunity to recombine over time.

Consequently, it was hypothesized that sexual recombination between distinct clonal lineages of *Plasmodium falciparum *parasites were altering the subpopulation structure and effectively maintaining the population-level diversity in this local population. Sexual recombination between distinct clones (clonets) was traced in this population using 14 microsatellite markers scattered across 10 chromosomes and then correlated with an independent antigenic marker, *Pf*msp1 Block 2 (*Pf*msp1-B2). The main allelic families of *Pf*msp1-B2 were found to correlate significantly with specific alleles from each of the microsatellite loci and were also found to have distinct microsatellite haplotype signatures. On a population-level, this resulted in the observation of a rigid clonal backbone that could be defined by *Pf*msp1-B2 main allelic families. However, this clonal population structure breaks down due to increased sexual recombination between constituents of complex infections that have been propagated together over time. The consequences of such sexual recombination could impact parasite evolution of drug resistance, host's susceptibility to infection, and even the expansion of new clonal variants.

## Methods

### Study design

DNA samples were selected from the Malaria Immunology and Genetics in the Amazon longitudinal cohort study in the community of Zungarococha, located in the San Juan District south of Iquitos, Peru. Details on the study site and design are described in [47]. In brief, this longitudinal study employs both active and passive case detection methods. Of the community of approximately 2,100 individuals, 1,945 individuals participated each year. In active case detection (ACD) there are at least six household visits during the seven month malaria season: one at the beginning of the malaria season, followed by four consecutive weekly visits during malaria season, and then one at the end of the malaria season.

Infections were defined as one or more malaria-positive sample point(s) during the one-month of follow-up (termed "infection-month). The sampling could, therefore, begin before the initial malaria positive sample or after, with an overall sampling time frame of less than or equal to 30 days (4 weeks of sampling) [41,46,47]. Infections were defined as complex when more than one allele was present within a single sample or over the infection-month as determined by *Pf*msp1-B2 genotyping. All blood samples, positive or negative by microscopy, underwent DNA extraction by Qiagen DNeasy Blood and Tissue Kits and were tested for presence of *Plasmodium *species using a nested multiplex PCR method targeting DNA encoding the small subunit ribosomal protein (ssrDNA) (Qiagen Inc., Valencia, CA) [48].

All passive case detection (PCD) samples collected from participating individuals were included in this study. Members of our field team, along with Ministerio de Salud (MINSA) personnel, staff the local health post, which services the community of Zungarococha. All individuals presenting with a fever are screened for malaria infection and treated if an infection is detected. These services are provided at no cost to the patient.

All protocols were reviewed and approved by the Institutional Review Boards at the University of Alabama at Birmingham (former institution), New York University School of Medicine, and MINSA. Written, informed consent/assent was obtained from all participants in this study.

### Microsatellite marker selection and amplification

Fourteen microsatellite markers were chosen from the microsatellite linkage map for amplification. Descriptive haplotypes were assembled for clones by concatenating the alleles detected at each microsatellite locus. Infections were classified as complex when two or more loci contained multiple alleles. Haplotype phasing was confirmed by cloning by limiting dilution (see below). All other methods are described in Branch et al. [46]. Summary information on microsatellite marker loci, number of alleles, and the amount of diversity per locus can be found in Additional file 1.

### Cloning by limiting dilution

Venous blood from *P. falciparum *positive individuals was collected in a BD Vacutainer^® ^(Becton-Dickinson (Becton, Dickinson and Co., Franklin Lakes, NJ, USA) and parasites were harvested and cryopreserved in a culture bank. A selection of 15 samples, determined to have two different alleles (30 different clones) based on *Pf*msp1-B2 genotyping, were cultured *in vitro*. At 3% parasitaemia, small-volume aliquots were passed to new culture flasks with fresh culture media to separate and expand the clones. At each passing, including day 0, a small aliquot of the culture was taken for DNA extraction and amplification by PCR for *Pf*msp1-B2 genotyping. Genotyping was performed at each passage until infections were passed as single clones over three passages. Additionally, microsatellite genotyping (using 14 microsatellites) was performed in triplicate on all samples before and after separating the clones by limiting dilution.

### *Pf*msp1-B2 genotyping analysis

PCR genotyping of *Plasmodium falciparum *merozoite surface protein 1 block 2 (*Pf*msp1-B2) was attempted on all available samples that were microscopy and/or PCR positive for *P. falciparum *between 2003 and 2007 (858 *P. falciparum *infection-months) [40,41]. Of the 858 infection-months, 814 were reproducibly genotyped. Previously reported genotyping methods were used for main allelic family analysis, using all permutations of primers to detect any allele families that were one allele type in the 5' direction and a different allele type in the 3' direction [40,41,44,49]. Of the 194 complex infections, as defined by *Pf*msp1-B2, there were only 46 (23.7%) instances when an infection was found to have more than one clone due to the detection of a different allele over time (≤ 30 days). These 46 infections were distributed over the five years of this study and did not bias the detection of complex infections.

### Genetic diversity data analysis

#### Cluster analysis

Structure 2.2, a Bayesian programme for grouping genetically related isolates, was used to define the population structure [50]. Methods for this analysis were previously reported in Branch et al. [46]. In brief, individual microsatellite haplotype profiles were assessed by the clustering algorithm and each haplotype was then given an estimated membership coefficient (*Q*) that translates to the fraction of relatedness (or ancestry) within/between each cluster [51]. *Q *is used to establish the clusters (*K*), which are reported as an admixture proportion. Clusteredness was used to determine the most accurate number of clusters representative of this population. Clusteredness calculations measure the average relatedness of the individual membership coefficients (*Q*) and estimate the extent to which individual infections belong to a single cluster, rather than to a combination of clusters [51]. For each value of *K *(pre-specified to equal between 1 and 10), the clustering algorithm was performed 5 times for 10,000 Monte Carlo Markov Chain iterations, followed by a burn-in period of 10,000 iterations. These results were then output and averaged to assess the clusteredness calculation for each value of *K*. Population differentiation was confirmed using F_st _and expected heterozygosity (H_e_) (Arlequin v3.5.1.2) [52]. No population structure was observed for any *K*-value above *K *= 10.

#### Minimum spanning network

A minimum spanning network (MSN) was developed in Network 4.5.1.0 using the microsatellite haplotype profiles [53]. For the MSN, the sample identifications were coded with a combination of the cluster-population (Clusters 1-6) and infection-type (complex or single-clone) information associated with each sample, allowing us to (1) visualize the relatedness between parasite infections, (2) examine how closely related the clusters were to one another, and (3) determine the frequency of complex versus single-clone infections in each cluster subpopulation.

#### Linkage disequilibrium

The standard index of association (I_A_^S^) was used to test for statistical independence of alleles at each of the 14 microsatellite loci for all subpopulations (LIAN v3.0) [54]. I_A_^S ^uses a robust Monte Carlo simulation that accounts for differences between the observed (*V_D_*) and expected (*V_e_*) mismatch variances of alleles, provides a measurement for linkage and tests for significance. If *V_D _*and *V_e _*are significantly different, then independent assortment of alleles cannot explain the observed diversity and the alleles are in disequilibrium.

#### Allelic and private allelic richness

Subpopulation diversity was calculated by measuring the allelic richness (mean number of alleles per locus) and private allelic richness (number of alleles private to a specific population) using a rarefaction approach for both measurements to model the data based on subpopulation sample-size (ADZE v1.0) [55].

Unless otherwise specified, all data analyses were performed using JMP^® ^v8.0.2 software (SAS Institute Inc., Cary, NC).

## Results

### Ecology of complex infections

#### Sample selection

Due to the single-copy nature of *Pf*msp1-B2, this gene was used to generically discriminate between single-clone and complex infections for all isolates collected in this study between 2003 and 2007. In total, 858 infection-months underwent genotyping by *Pf*msp1-B2; 814 of these were reproducibly genotyped and served as the baseline for genetic diversity (Table 1). Based on the proportion of different infection-types (single-clone versus complex infections) detected during each year of this study, a random sample subset was selected to undergo microsatellite genotyping. Of the 814 infection-months genotyped by *Pf*msp1-B2, 243 were selected for microsatellite analysis (Table 2). Due to the infrequent detection of infections with more than two clones, infections with more than one clone were defined "complex", while infections with only one clone were defined as "single-clone".

**Table 1:** *Pf*msp1-B2 genotyping results for all samples collected between 2003 and 2007

Infection-type	2003	2004	2005	2006	2007
Single infections	89	205	192	96	38
Complex infections	7	40	85	41	21

Interfamily	4	29	73	38	20
Intrafamily	3	11	12	3	1

**Table 2:** Samples selection breakdown

Method	Infection-types	2003	2004	2005	2006	2007
***Pf*msp1-B2**	Single infections	12	42	74	48	13
	Complex infections	4	11	18	13	8
		
	Interfamily	3	7	13	12	8
	Intrafamily	1	4	5	1	0
	**Total number of clones:**	21	66	113	76	29

**Microsatellite genotyping**	Single infections	7	41	68	52	13
	Complex infections	9	12	24	9	8
	**Total number of clones:**	25	67	119	73	29

#### *Pf*msp1-B2 analysis

**Figure 1:**
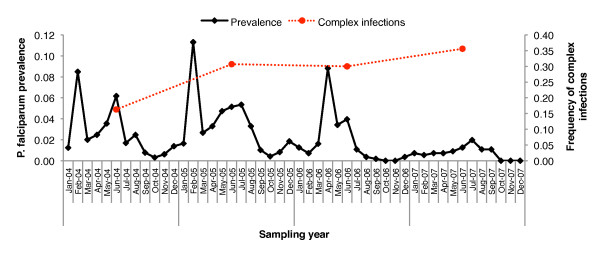
***Plasmodium falciparum *infection prevalence and frequency of complex infections over time**. Despite the declining infection prevalence over time (black line), the proportion of complex to single-clone infections increases (red, dotted line).

The proportion of complex versus single-clone infections for each year was calculated (excluding year-2003, due to collection beginning after the start of malaria season). There was a 2.18-fold increase in the proportion of complex infections versus single-clone infections, increasing from 16.3% in 2004 to 35.6% in 2007 (p < 0.001, χ^2^). Next, these proportions were correlated with respective infection prevalence rates for each year of this longitudinal cohort study. Between 2004 and 2007, the infection prevalence decreased by 61.3%, from 0.31 infections/person/year in 2004 to 0.12 infections/person/year in 2007 (p < 0.0001, χ^2 ^logistic regression whole model test). Despite the decreased infection prevalence over time, the proportion of complex infections increased significantly (Figure 1).

This increased detection of complex infections over time is not due to sampling bias. The average number of complex infections detected through passive case detection (PCD) is significantly lower than those detected though active case detection (ACD) for each year of this study between 2004 and 2007 (p < 0.001, χ^2^). Also, the distribution of complex infections over time within each of the four networked-villages was proportionate; there was no indication that one or more of the villages may be defined by having a greater transmission of complex infections.

**Figure 2:**
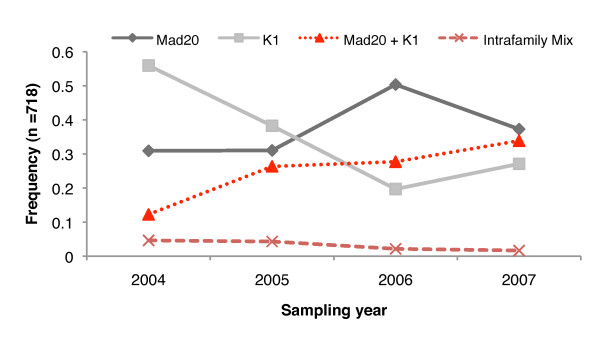
**Distribution of *Pf*msp1-B2 allelic families over time**. Despite a shift in the dominant allelic families circulating in the population, the increased proportion of complex to single-clone infections is attributable to complex infections containing both Mad20 and K1 clonal variants.

The number of interfamily complex infections (Mad20 and K1) was significantly greater than intrafamily complex infections (only K1 or only Mad20 alleles) for each year between 2004 and 2007, increasing slightly each year from 72.5% of all complex infections in 2004 to 95.2% in 2007 (p < 0.034, Fisher's Exact) (Figure 2). This is important and relevant because the increased proportion of complex to single-clone infections is attributable to complex infections containing both Mad20 and K1 clonal variants (Figure 2).

#### Microsatellite analysis

Alleles determined by each of the 14 microsatellite loci were assembled into a single haplotype for each clone within an isolate. To confirm the accuracy of microsatellite haplotype phasing, 30 parasite clones were isolated from complex infections by *in vitro *culture, using cloning by limiting dilution methods (See methods). Microsatellite genotyping was performed before and after isolating the 30 clones. Twenty-three of the 30 clones were identical to the microsatellite haplotypes compiled without cloning out by limiting dilution. Of the seven discrepant microsatellite haplotypes, five had discrepancies in one locus (92.9% match with non-isolated haplotype) and two had discrepancies in two loci (85.7% match with non-isolated haplotype). However, the correct locus was always observed in at least one or two of the genotyping replicates, but never all three as was observed in the 23 clones.

In total, there were 313 clones detected in the 243 infection-months selected for microsatellite analysis; 62 of these infection-months were determined to be complex infections. These results were directly correlated to the *Pf*msp1-B2 genotypic classification of infection-type and a *kappa *test was used to assess the strength of agreement between the proportions of complex versus single-clone infections defined by both genotyping methods.

The average COI (total number of clones divided by the total number of infection-months) differed slightly between the *Pf*msp1-B2 (305 clones detected in 243 infections, COI = 1.26) and microsatellite genotyping (313 clones detected in 243 infections, COI = 1.29) methods (Table 2). Of the 243 infection-months selected, 54 of these were defined as complex by *Pf*msp1-B2 genotyping (43 interfamily and 11 intrafamily), while 62 were defined as complex by microsatellite genotyping (14 markers scattered over 10 different chromosomes) (Table 2). Detecting an increased number of complex infections using the microsatellite assay was anticipated due to the use of multiple loci. The *kappa *value was equal to 0.523 (95% CI = 0.447-0.598), indicating moderate strength of agreement between these genotyping methods.

### Subpopulation structure

#### Microsatellite haplotype population structure analyses

Structure v2.2 [50] was used to describe the subpopulation structure based on the 14 microsatellite loci haplotypes for all clones (N = 313) from all 243 infections in this sample-set. A *post-hoc *clusteredness calculation was performed to determine accuracy of predetermined number of clusters (*K*) [51]. The highest clusteredness value over 10 simulations showed *K *equal to 6, with a clusteredness value equal to 0.81. Arlequin v3.5.1.2 [52] was used to test the population differentiation (F_st_) between these clusters. Each of the six clusters was found to represent independent subpopulations (p < 0.01, Pairwise F_st_). On a spatial and temporal level, these six cluster subpopulations were maintained in all four-networked villages across each year of this study.

**Figure 3:**
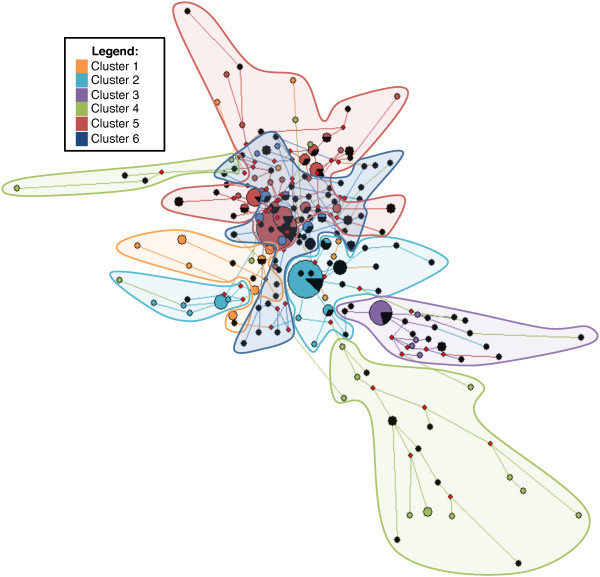
**Minimum Spanning Network (MSN) with cluster analysis superimposed**. The Clusters are differentiated by colors: orange = C1, light blue = C2, purple = C3, green = C4, red = C5, and dark blue = C6. Black dots are constituents of complex infections and red dots are putative progenitors. Figure backbone is the MSN with cluster-colored circles representing individual infections. Circle size is proportionate to the number of infections with the same MS haplotype. Cluster-colored clouds superimposed on the MSN are indicative of cluster-grouping. Distance between circles is indicative of the number loci polymorphisms.

Network v4.1.5.0 [53] was used to further describe the parasite subpopulation structure based upon the microsatellite loci haplotypes. The resulting minimum spanning network (MSN) in Figure 3 is coded by cluster affiliation (Clusters 1-6) to visualize relatedness between subpopulations. There was minimal interconnectedness between Clusters 1-5, while Cluster 6 was central in the MSN and had widespread connections within all Clusters, except Cluster 3. This indicates an increased relatedness between Cluster 6 and Clusters 1, 2, 4, and 5. Additionally, Cluster 6 showed an increased number of theoretical progenitors, compared with all other clusters (Figure 3).

### Recombination as a factory for diversity

#### Underrepresented identical haplotypes

For each cluster subpopulation the number of haplotypes present, expected heterozygosity (H_e_), allelic richness, and the private allelic richness were considered (Table 3). Of the 313 clones identified in this sample-set, 197 (62.9%) of these were defined by unique haplotypes. This excess of unique haplotypes is further observed within each of the six cluster subpopulations. Unique haplotypes in Clusters 1, 3, 4, and 6 exceed 70% of the total number of clones within each cluster, while Clusters 2 and 5 have lower proportions of unique haplotypes, but still exceed 46% of the total number of clones within each cluster (Table 3). The lower proportion of unique haplotypes in Cluster 2 and 5 were due to both clusters having a single dominant haplotype that is repeatedly detected over time. In Cluster 2, 31 of 63 (49.2%) clones share the dominant haplotype, while 40 of 117 (34.1%) clones share the dominant haplotype in Cluster 5.

**Table 3:** Measurements and indices of subpopulation genetic diversity

Cluster	No. of samples	No. of haplotypes	Genetic diversity			Linkage disequilibrium	
			
			H_e_	Allelic richness	Private allelic richness	I_A_^S^	p-value
1	30	22	0.400	3.79	0.228	0.036	< 0.004
2	63	29	0.145	3.50	0.092	0.150	< 0.001
3	29	25	0.220	2.79	0.080	0.081	< 0.001
4	34	32	0.593	4.71	0.800	0.066	< 0.001
5	117	54	0.209	3.86	0.133	0.115	< 0.001
6	40	35	0.314	3.57	0.180	0.013	0.072

All Clusters	313	197	0.439	6.07	na	0.103	< 0.001

Interestingly, in spite of the high frequency of unique haplotypes within each cluster, there were relatively low levels of genetic diversity within each of these subpopulations. H_e _(Arlequin v3.5.1.2) was used to measure the genetic diversity based on the relative frequencies of the different alleles within each of the subpopulations. There was low H_e _in Clusters 1, 2, 3, 5, and 6, with a range of 0.15 to 0.40. To further investigate the subpopulation diversity, allelic richness (mean number of alleles per locus) and private allelic richness (number of alleles private to a specific population) were measured using a rarefaction approach to standardize and model the data based on subpopulation sample-size (ADZE v1.0). After correction for sample-size, Clusters 1, 2, 3, 5, and 6 averaged 4 alleles per locus, with approximately 23% of the alleles detected within each of these five subpopulations identified as private alleles. Cluster 4 deviates from the trend observed in Clusters 1, 2, 3, 5, and 6, with a moderately higher H_e _equal to 0.593 and an allelic richness equal to 4.71 alleles per locus with approximately 80% of the alleles detected identified as private alleles (Table 3).

**Figure 4:**
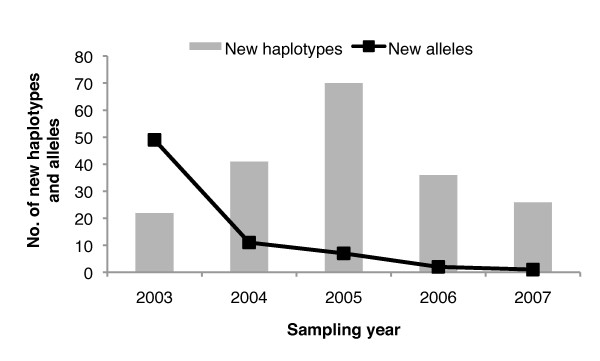
**Frequency of new alleles and haplotypes over time**. The sampling epoch (2003-2007) is indicated on the x-axis, number of new haplotypes on the primary y-axis, and number of new alleles on the secondary y-axis. The detection of new haplotypes over time was not due to the detection of new alleles over time. In fact, > 65% of all of the alleles detected across all five years of this study were detected in 2003, the first year of this study.

In summary, identical haplotypes were underrepresented within this population even though the genetic diversity is quite limited on a subpopulation level. Such a dynamic is only possible if the number of alleles, and not the combination of alleles, is limited. This dynamic supports recombination as a potential contributor to the maintenance of genetic diversity within this population. Further, in Figure 4 the number of new haplotypes versus the number of new alleles detected in all subpopulations between years 2003 and 2007 is compared. In total, 70 different alleles were detected across the 14 microsatellite markers selected for this study. The robust sampling strategy of this study (passive and active case detection) identified 70.0% (n = 49) of these alleles in the first year of collection (year-2003). There was a gain of 25.7% (n = 18) of the total number of alleles identified by 2005, and a gain of only 4.3% (n = 3) by year 2007 (Figure 4). The appearance of new haplotypes is not associated with the appearance of new alleles over time. The majority of the new alleles detected over time in this study were minor in frequency. Additional file 2 illustrates the frequency of each allele detected in this study over time.

#### Linkage disequilibrium

The standard index of association (I_A_^S^) for each cluster was measured to test for statistical independence amongst alleles (independent assortment of alleles) at each of the 14 microsatellite loci (Table 3). Based on this method, Clusters 1, 2, 3, 4, and 5 were in linkage disequilibrium (LD) (p < 0.005, 10000-step Markov Chain analysis, one-tailed t-test, LIAN v3.0), while break down in LD was observed in Cluster 6 (p = 0.072, 10000-step Markov Chain analysis, one-tailed t-test, LIAN v3.0) (Table 3). This decay in LD associated with Cluster 6 is indicative of an independent assortment of alleles, which is a signature of this subpopulation approaching linkage equilibrium by undergoing sexual recombination.

#### Breakdown in correlation between independent sets of genetic markers in the complex infection subpopulation

Next, *Pf*msp1-B2 genotypes were correlated to the six cluster subpopulations as determined by using the 14 microsatellite loci. Using a probability model, the expected *Pf*msp1-B2 genotype frequencies were compared to the observed genotype frequencies in all six clusters. With the exception of Cluster 6, one *Pf*msp1-B2 main allelic family (K1 or Mad20), and often one specific *Pf*msp1-B2 allele (repeat length), significantly correlated with the clones defining each cluster (p < 0.001, χ^2^). Clusters 1 and 5 were significantly comprised of single-clone Mad20 alleles, while Clusters 2 and 4 were significantly comprised of single-clone K1 alleles. Cluster 6, the exception to this population structure, was significantly comprised interfamily complex infections (K1 plus Mad20), and also had the highest number of interfamily complex infections of all six subpopulations (p = 0.0044, Fisher's exact). The subpopulation structure of Cluster 6 may explain the previously observed decay in LD associated with this cluster, as this cluster may have evolved from recombination events between the two most frequently observed genotypes in this population.

#### Appearance of recombined haplotypes over time

**Figure 5:**
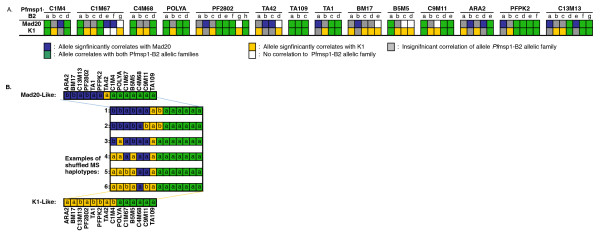
**Examples of observed progenitors and recombined microsatellite haplotypes. A) Correlation of *Pf*msp1-B2 allelic families with individual microsatellite alleles in the population**. Each microsatellite locus is identified on the horizontal, while the correlated *Pf*msp1-Block 2 main allelic family is indicated on the vertical. Letters under each microsatellite locus are indicative of individual alleles (repeat-lengths) ordered sequentially by frequency within the population. Colored blocks are used to indicate a correlation with a specific *Pf*msp1-Block 2 main allelic family and are identified in the legend. **B) Examples of potential recombinants between two single-clone lineages**. Illustrates the two most frequent single-clone infections that may have given rise to these six examples of complex infections (shown on the inlay) haplotypes by genomic shuffling (putative progenitor clones).

To further investigate the evolution of Cluster 6, recombination between microsatellite alleles was investigated. Due to the significant correlation between the cluster subpopulations and the *Pf*msp1-B2 genotypes, microsatellite haplotypes were identified by their *Pf*msp1-B2 genotype: K1, Mad20, or K1 plus Mad20. In total, 57.1% (40 of 70) of the microsatellite alleles were significantly correlated to either the K1 or Mad20 allelic families (Figure 5A). This enabled a straightforward association of K1 and Mad20 alleles to specific microsatellite alleles identified in the population. On the most basic level, this showed that K1 and Mad20 alleles might be in disequilibrium with specific microsatellite alleles, which has resulted in a rigid clonal backbone in this population.

However, this correlation also enabled the meticulous tracing of sexual recombination across the 14 microsatellite loci. To trace possible recombination events, the two most frequent microsatellite haplotypes observed over time (defined by either a K1 or Mad20) were selected as the progenitors and then potential recombinants were identified in within the natural population. Six examples of potential recombinants that may have arisen from recombination between the Mad20- and K1-like dominant haplotypes are illustrated in Figure 5B. There is clear evidence for a breakdown in the correlation between these two independent genetic markers, likely due to recombination between clones alternatively identified by these two main allelic families.

**Figure 6:**
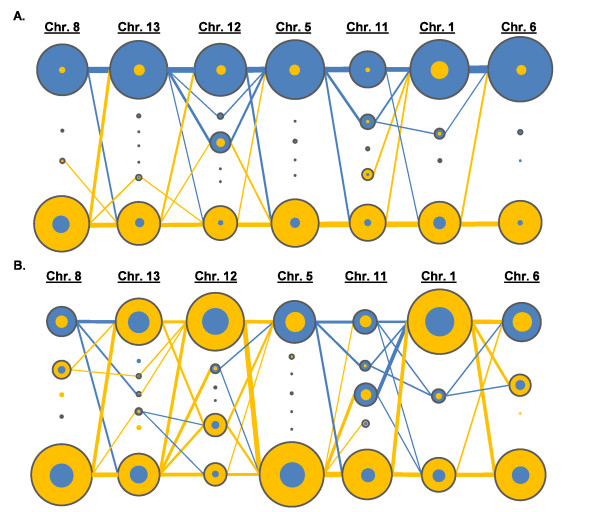
**Model illustrating the clonal backbone of single-clone infections (A) and homogenization between constituents of complex infections (B)**. Each vertical column of circles represents a specific microsatellite locus (seven total for both A and B) and the number of independent circles within each vertical column represents the number of alleles detected within that particular locus. Blue and yellow coloring is indicative of a correlation with either Mad20 (blue) or K1 (yellow) *Pf*msp1-Block2 main allelic families, respectively. The size of the colored circles represents the number of infections indentified by a particular allele that was associated with either Mad20 or K1. When circles are superimposed, this shows that alleles were correlated (with varying proportions based on circle-size) to both Mad20 and K1. Lines between loci indicate the number of instances (differentiated by the line thickness) that one allele paired with another allele in the population. **A) **Illustrates the clonal backbone present within single-clone infections, indicated by having a predominantly blue (Mad20) clonal family and a predominately yellow (K1) clonal family. This is further supported by a fewer number of lines between loci pairs and predominantly clonal haplotypes. **B) **Illustrates a breakdown in linkage between loci pairs, with an increased number of loci pairs and a relatively equal distribution of clones correlated to each *Pf*msp1-B2 main allelic family causing the disappearance of the clonal backbone.

The correlation between dominant *Pf*msp1-B2 alleles and microsatellite markers was mapped to visualize the breakdown in the clonal backbone. A breakdown in this correlation would indicate homogenization between these genomes. For analytical ease, only seven of the most highly correlated microsatellite loci were selected for this study. Indeed, there was a discrete clonal backbone in the single-clone infections that was highly linked with the two *Pf*msp1-B2 main allelic families, K1 and Mad20 (Figure 6). However, in the interfamily complex infections the clonal backbone disappears as the correlation between individual microsatellite alleles and *Pf*msp1-B2 main allelic families breaks down. Figure 6 clearly illustrates a population with complex infections that have undergone many recombination events, such that the overall population structure of these infections has become markedly more homogenous.

Finally, this genome homogenization was examined over time. Populations were divided into "early years" (2003-2005) and "late years" (2006-2007). For complex infections, most of which belonged to Cluster 6, there was a 36.1% (13 new loci pairs and 36 repeated loci pairs) increase in the number of new loci combinations detected over time. The frequency of these new allelic combinations became rapidly integrated into the natural population, defining 34.3% of the complex infections detected between 2006 and 2007. In contrast, there was only a 16.0% (8 new loci pairs and 50 repeated loci pairs) increase in the number of new loci combinations observed in single-clone infections. These new combinations only defined 6.7% of the single-clone infection population detected between 2006 and 2007. The frequency of new loci combinations emerging in this study over time was significantly higher in complex than single-clone infections (p < 0.0001, χ^2^).

The increased combination of loci pairs and their increased frequency over time is consistent with recombination between constituents of complex infections, a distinct evolutionary signature from that of the single-clone infections. The detection of new alleles over time cannot explain the rise of new combinations of alleles, as there were only three new alleles detected at an exceedingly low frequency in the population between 2006 and 2007. It is clear that these new combinations have arisen from recombination and not by the introduction of new alleles.

## Discussion

In this study, the evolutionary impact of multiple clone, complex infections within a local and low transmission population in the Peruvian Amazon between 2003 and 2007 was investigated. Previously, researchers have found that in exceedingly low transmission regions, such as the Karen Refugee Camp in Thailand, there were more complex infections than expected by chance [39]. Though unexpected in low transmission settings, a niche with a high frequency of complex infections increases the potential for sexual recombination between genetically distinct clones within local populations. The formation, propagation, and ultimately the survival of complex infections in low transmission regions are of particular interest because these types of infections provide an increased opportunity for genetic diversity to be maintained through recombination. A study of this nature is not feasible in high transmission regions because the complexity of infection is generally high due to overlapping infections and virtually impossible to separate infections into individual clonal constituents.

An earlier study, which examined the ecology of complex infections in this cohort from 2003-2004, found that complex infections within humans were not formed by an accumulation of clones by superinfection, but rather, complex infections were being propagated as a single unit during a blood meal [41]. In this study, *Pf*msp1-B2 was genotyped from all *P. falciparum *positive samples collected between 2003 and 2007 to determine the frequency of complex infections over time. This finding was supported by the sampling and genotyping strategies used in this cohort study. All samples collected, regardless of a positive or negative diagnosis by microscopy undergo species-specific PCR. Due to the fact that many of infections in this community are asymptomatic, it is particularly important to consider the possibility of subpatent carriers. For this reason, sampling occurs over a one-month time-period and final genotypes for all infections are concatenated from multiple sample collections (4-6 collections over 4-weeks), rather than a single time point; this time-point genotyping is referred to as infection-months in this manuscript.

When the longitudinal genotyping data was correlated to infection prevalence data between years 2003 and 2007, the frequency of complex infections were found to increase significantly from 16.3% in 2004 to 35.6% in 2007 in spite of a substantial decrease in the infection prevalence from 0.31 infections/person/month in 2004 to 0.12 infections/person/month in 2007, while the (p < 0.001, χ^2^). This dynamic does not support an environment whereby complex infections were being generated by superinfection, but rather it provides additional evidence that the complex infections in this low transmission region were being propagated as single evolutionary-units.

The increase in complex infections relative to the single-clone infections was striking given the overall decrease in infection prevalence over the years of this study (Figure 1). Two different scenarios can be speculated to explain this increase over time that is not related to the human host immune response. One possible explanation is that the increase in complex infections over time is an ecological phenomenon, rather than differential survival probabilities of complex versus single-clone infections. For example, it is possible that there is an ecological niche that maintains the transmission of complex infections over time, like mosquito breeding sites or potential reservoirs. However, the distribution of complex infections over time within each of the four networked-villages was proportionate, with no indication that one or more of the villages have a greater frequency of complex infections. Another possible explanation is that complex infections are more readily transmitted from the human host to the mosquito host [56-68]. This may be linked with an environmental stimulus that signals an increased production of gametocytes when parasites are under stress [59-63], but it is still unclear whether or not this may also be linked to disease outcome within the human host.

Regardless of why complex infections were increasing in the population, the result of such infections being propagated longitudinally would be a reduced chance of encountering the same genotype over successive infections due to increased opportunities for recombination [64]. The hypothesis in this study was that despite a superficial clonal population structure, sexual recombination between distinct clonal lineages of *P. falciparum *parasites is altering the sub-structure of this population. To directly test this hypothesis, previously defined criteria for distinguishing between clonal and sexual populations was used [25].

Using microsatellites to longitudinally characterize the population and within-host level genetic diversity, it is clear that the parasite population can be divided into six distinct cluster subpopulations that were maintained over time in this study. The frequency of identical and unique microsatellite haplotypes were then investigated within the total population and within each of the six cluster subpopulations. Of the 313 clones in the total population, there were 197 unique haplotypes observed (Table 3). These unique haplotypes were not isolated to a particular subpopulation, but rather were found across each of the six subpopulations (clusters) of related clones. Clusters 1, 3, 4, and 6 have unique haplotypes that exceed 70% of the total number of clones within each cluster, while Clusters 2 and 5 have a lower proportion of unique haplotypes, but still exceed 46% (Table 3). This contradicts what is expected in the null hypothesis.

The excess of unique haplotypes was further investigated by measuring the H_e_, allelic richness, and private allelic richness within each of the six cluster subpopulations (Table 3). These analyses revealed that the number of alleles is limited, while the combination of alleles (which comprise the haplotypes), is not limited. This indicated that a breakdown in linkage between the loci could help to explain the excess of unique haplotypes. Next, a test for independent assortment of alleles was performed using the standard index of association (I_A_^S^). This test indicated a significant decay in LD restricted to Cluster 6 (Table 3). On a parasite level, predicting the genetic exchange during one round of sexual recombination is at best an educated guess, but over ten years of persisting recombination, it is virtually impossible to do, even with sophisticated models. Due to the significant decay in LD associated with Cluster 6, this cluster became a model for recombination in this study; however, Cluster 6 was not the only cluster where recombinants were identified.

To further consider the breakdown of LD in Cluster 6, the correlation between the microsatellite haplotypes and the single-copy antigenic locus *Pf*msp1-B2 was examined. Using *Pf*msp1-B2 as a generic and independent genetic marker permitted a broad characterization of the genome and further insight into the ecology of the infections analyzed. Five of these subpopulations (Cluster 1-5) were significantly comprised of single-clone infections that significantly correlated with specific *Pf*msp1-B2 main allelic family genotypes (either K1 or Mad20), while Cluster 6 was significantly comprised of complex infections that were a mixture of *Pf*msp1-B2 main allelic family genotypes (K1 and Mad20). A breakdown in linkage could be readily observed by considering the linkage in the single-clone (K1 or Mad20) versus complex clone infections (K1 and Mad20) (Figure 5; Figure 6). The population structure of the single-clone infections define a clonal backbone with strong linkage to the antigenic individual *Pf*msp1-B2 genotypes, while the complex infections undergo apparent recombination, which breaks down the linkage between alleles in this backbone. The result of this is an increased number of unique haplotypes over time (Figure 4). Further, it was possible to single-out potential progenitors of recombined clones, which strengthens the evidence of sexual recombination contributing to the parasite genetic diversity within this population (Figure 5).

Repeated or recursive recombination naturally results in the homogenization of different parasite clones or strains, unless there is some barrier to recombination that maintains a clonal backbone structure. Although microsatellite markers are considered neutral, there might be linkage with antigens scattered across the genomes. If this is the case, the recombination may breakdown linkage between antigen encoding genes, which is particularly useful if the human host is immune to reinfection by recently encountered genotypes [34,64-72]. Others have suggested that recombination is favorable in changing environments, like parasite-host infections that are often modulated by the immune response [34,72]. However, the observation of recombined microsatellite haplotypes in this study could also be due to one particular locus under selection. That is, it is possible that a particular mutation/allele or combination of alleles, which may have been fixed in the population during the propagation of complex infections over time, resulting in a population with a slight selective advantage [65-68,73].

In the most practical context, this study shows that genetic diversity can increase even when there is low transmission. This scenario is likely to be reencountered in the future as the current *P. falciparum *elimination efforts are effectively reducing clinical malaria in many countries across the world. The expansion of recombined clones within a local population could increase host susceptibility to infection, and also provide more opportunities for the evolution of drug resistance.

## Conclusions

The aim of this study was to consider the impact that multi-clone, complex infections have on a parasite population structure in a low transmission setting. Contrary to conventional low transmission models, this study provides evidence of a parasite population structure that is superficially defined by a clonal backbone. Sexual recombination does occur and even arguably is responsible for maintaining the substructure of this population. The maintenance of genetic diversity in this population is likely a consequence of the long-term propagation of complex infections in this region. The fact that these recombinants were surviving in this population, despite a decreasing prevalence rate, raises an important question on the fitness of these parasites and/or the accumulation of clones within one infection.

## Conflicts of interests

The authors declare that they have no competing interests.

## Authors' contributions

PS and OB designed research; PS and LS performed research; PS and OB analysed data; PS and OB wrote the paper. All authors have reviewed and approved this manuscript.

## Supplementary Material

Additional file 1 **Microsatellite loci, alleles, and diversity**. Description of the microsatellite loci used, the number of alleles detected, and the expected heterozygosity of each locus within all samples used in this study. Click here for file

Additional file 2 **Longitudinal frequency of alleles detected in study**. Illustrated here are the frequencies of each allele, within each locus, over the five years of this study. Different shades of gray indicate the proportion of a detected allele with respect to the locus and the year of collection. Click here for file
