# Supplementary material for: Sexual recombination is a signature of a persisting malaria epidemic in Peru
Source: Malar J. 2011 Oct 31;10:329. doi: 10.1186/1475-2875-10-329 (PMC3231964; doi:10.1186/1475-2875-10-329)
Supplement: Additional file 1 — Microsatellite loci, alleles, and diversity. Description of the microsatellite loci used, the number of alleles detected, and the expected heterozygosity of each locus within all samples used in this study. [file 1475-2875-10-329-S1.DOC]

| **Additional file 1.** | | | |
| --- | --- | --- | --- |
| **Microsatellite loci, alleles, and diversity** | | | |
| **MS Marker** | **Chr.** | **No. of alleles** | **H_e_** |
| C1M4 | 1 | 4 | 0.567 |
| C1M67 | 1 | 7 | 0.255 |
| B5M5 | 3 | 4 | 0.229 |
| C4M69 | 4 | 4 | 0.388 |
| POLYA | 4 | 4 | 0.352 |
| PF2802 | 5 | 8 | 0.585 |
| TA42 | 5 | 4 | 0.205 |
| BM17 | 6 | 4 | 0.606 |
| TA109 | 6 | 3 | 0.338 |
| TA1 | 6 | 4 | 0.598 |
| C9M11 | 9 | 5 | 0.183 |
| ARA2 | 11 | 5 | 0.651 |
| PFPK2 | 12 | 6 | 0.617 |
| C13M13 | 13 | 7 | 0.631 |
