# Supplementary figures and images for: Sexual recombination is a signature of a persisting malaria epidemic in Peru
Source: Malar J. 2011 Oct 31;10:329. doi: 10.1186/1475-2875-10-329 (PMC3231964; doi:10.1186/1475-2875-10-329)

### Longitudinal frequency of alleles detected in study

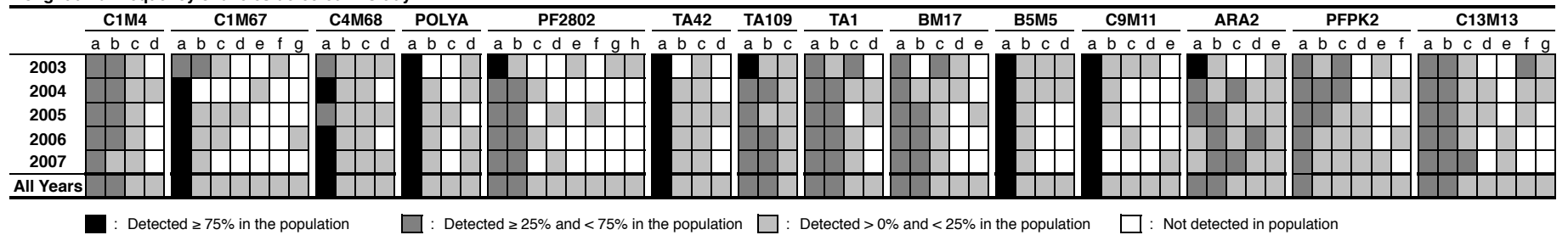

Supplement: Additional file 2 — Longitudinal frequency of alleles detected in study. Illustrated here are the frequencies of each allele, within each locus, over the five years of this study. Different shades of gray indicate the proportion of a detected allele with respect to the locus and the year of collection. [file 1475-2875-10-329-S2.PDF]
